# Nanosecond pulsed electric field suppresses growth and reduces multi-drug resistance effect in pancreatic cancer

**DOI:** 10.1038/s41598-023-27605-4

**Published:** 2023-01-07

**Authors:** Wojciech Szlasa, Olga Michel, Natalia Sauer, Vitalij Novickij, Damian Lewandowski, Paulina Kasperkiewicz, Mounir Tarek, Jolanta Saczko, Julita Kulbacka

**Affiliations:** 1 Faculty of Medicine, Wroclaw Medical University, Wroclaw, Poland; 2 Department of Molecular and Cellular Biology, Faculty of Pharmacy, Wroclaw Medical University, Wroclaw, Poland; 3 Department of Cytobiochemistry, Faculty of Biotechnology, University of Wrocław, Wroclaw, Poland; 4 Faculty of Pharmacy, Wroclaw Medical University, Wroclaw, Poland; 5 Institute of High Magnetic Fields, Vilnius Gediminas Technical University, Vilnius, Lithuania; 6 Department of Immunology, State Research Institute Centre for Innovative Medicine, Santariškių 5, 08410 Vilnius, Lithuania; 7 Department of Animal Developmental Biology, Faculty of Biological Sciences, University of Wroclaw, Wroclaw, Poland; 8 Department of Chemical Biology and Bioimaging, Faculty of Chemistry, Wroclaw University of Science and Technology, Wroclaw, Poland; 9 Université de Lorraine, CNRS, LPCT, 54000 Nancy, France

**Keywords:** Biophysics, Cancer, Cell biology, Drug discovery, Medical research, Molecular medicine, Oncology

## Abstract

Nanosecond pulsed electric fields (nsPEF) have been shown to exert anticancer effects; however, little is known about the mechanisms triggered in cancer cells by nanosecond-length pulses, especially when low, sub-permeabilization voltage is used. In this study, three human pancreatic cancer cell lines were treated with nsPEF and molecular changes at the cellular level were analyzed. Further, we assessed the efficacy of paclitaxel chemotherapy following nsPEF treatment and correlated that with the changes in the expression of multi-drug resistance (MDR) proteins. Finally, we examined the influence of nsPEF on the adhesive properties of cancer cells as well as the formation and growth of pancreatic cancer spheroids. Cell line response differed with the application of a 200 ns, 100 pulses, 8 kV/cm, 10 kHz PEF treatment. PEF treatment led to (1) the release of microvesicles (MV) in EPP85-181RDB cells, (2) electropermeabilization in EPP85-181RNOV cells and (3) cell shrinkage in EPP85-181P cells. The release of MV’s in EPP85-181RDB cells reduced the membrane content of P-gp and LRP, leading to a transient increase in vulnerability of the cells towards paclitaxel. In all cell lines we observed an initial reduction in size of the cancer spheroids after the nsPEF treatment. Cell line EPP85-181RNOV exhibited a permanent reduction in the spheroid size after nsPEF. We propose a mechanism in which the surface tension of the membrane, regulated by the organization of actin fibers, modulates the response of cancer cells towards nsPEF. When a membrane’s surface tension remains low, we observed some cells form protrusions and release MVs containing MDR proteins. In contrast, when cell surface tension remains high, the cell membrane is being electroporated. The latter effect may be responsible for the reduced tumor growth following nsPEF treatment.

## Introduction

Efficient treatment of pancreatic cancer remains one of the greatest challenges of current oncology^1^. American Cancer Society estimates over 60 000 new pancreatic cancer patients were diagnosed in 2021. Pancreatic cancer treatment protocol depends on the clinical stage of the disease^2,3^. For the locally advanced pancreatic cancer, the most common treatment options are surgery and chemoradiation therapy^4^. When the cancer mass infiltrates the aorta, palliative treatment is introduced to reduce the tumor size and symptoms. In metastatic or recurrent pancreatic cancer, palliative chemotherapy combined with novel targeted therapeutics is indicated^4^. Selective kinase inhibitors remain the most promising option for patients diagnosed with advanced stage of the disease^5–7^. Unfortunately, due to the low tumor vascularization and efficient mechanisms of multidrug resistance (MDR) of pancreatic cancer cells, chemotherapy is characterized by low effectiveness^8–10^. The lack of prominent immunotherapy and low efficacy of chemotherapy underscore the need for progress in the search for more effective therapy alternatives^2,4^.

Treatment with pulsed electric field (PEF) has been proven effective against numerous neoplasms, like melanoma or prostate cancer^11–13^. PEFs have been shown to yield various effects depending on applied parameters (voltage, pulse duration, frequency of pulses delivery and number of pulses), we may describe various utilities of PEF. When voltage exceeds the electroporation threshold, reversible pores are formed in the cell membranes^14^. Simultaneous administration of low-voltage PEFs and cytostatic drugs enhance drug delivery to tumor cells^15^. When the electric field greatly exceeds the electroporation threshold, the process leads to irreversible electroporation (IRE)^16^. In this process, the cancer cell membrane permanently loses its integrity and a leakage of a cytoplasmic content is observed in the extracellular space. Even though IRE was initially thought to induce necrosis exclusively, current literature shows that the efficacy of chemotherapy is enhanced when combined with gemcitabine, bleomycin and calcium ions^17–19^. Changes in the pulse duration also led to different response of cancer cells towards PEF. Microsecond pulses are often used to permeabilize cell membrane and nanosecond pulses are applied to induce cytoskeleton disorganization and cell damage^20,21^. Pulses delivered with high frequency (MHz) are perceived by cells as a single cumulative pulse^22^ and low-frequency pulses are perceived as the series of pulses^21^. The variety of effects evoked in cells by PEFs requires researchers to optimize electroporation parameters to reach the desired biological effects.

The most important factors considered in pancreatic cancer treatment are local cancer growth, invasions in the surrounding tissues and a moderate response to chemotherapy^23^. Tumor microenvironment was also demonstrated to play important role in pancreatic cancer carcinogenesis and should not be disregarded in the anticancer regimen^24^. Nanosecond pulses have already been used to induce cell death or sensitize cells towards anticancer therapy^21,25,26^. It was shown that nsPEF may lead to the alteration of an antigens’ profile of cancer cell membranes^27^; however, to our knowledge, there is no research demonstrating nsPEF effects on the expression of MDR proteins.

This study aims to analyze the effects of nsPEF on the MDR effect, cell adhesion and the growth suppression of 3D pancreatic tumor model. For the experiments we used three pancreatic cancer cell lines with different profiles of drug resistance: EPP85-181RDB (resistant to daunorubicin), EPP85-181RNOV (multidrug resistant) and EPP85-181P (parental). Cells were treated with 100 pulses of 200 ns duration, delivered at 10 kHz frequency, with the electric field intensity of 8 kV/cm. Immediately following pulse delivery, cells were subjected to structural analysis with a holotomography microscope. Simultaneously we examined cell membrane permeabilization to Yo-Pro-1 fluorescent dye induced by PEF treatment. Further, we analyzed the organization of actin fibers in cells under a confocal microscope. The next part of the study aimed to assess the effects of nsPEF on the expression of MDR proteins. We analyzed the changes in p-glycoprotein (P-gp / CD243) and the low-density lipoprotein receptor-related protein (LRP, CD91) following cell stimulation with nsPEF and its implication in paclitaxel chemotherapy in vitro. A confocal microscope was used to examine pan-cadherin and N-cadherin immunoreactivity after nsPEF treatment. These results were compared to data on formation and growth of pancreatic cancer spheroids in order to assess cell adhesive properties.

## Results

### Effects of nsPEF on pancreatic cancer cells

**Figure 1:**
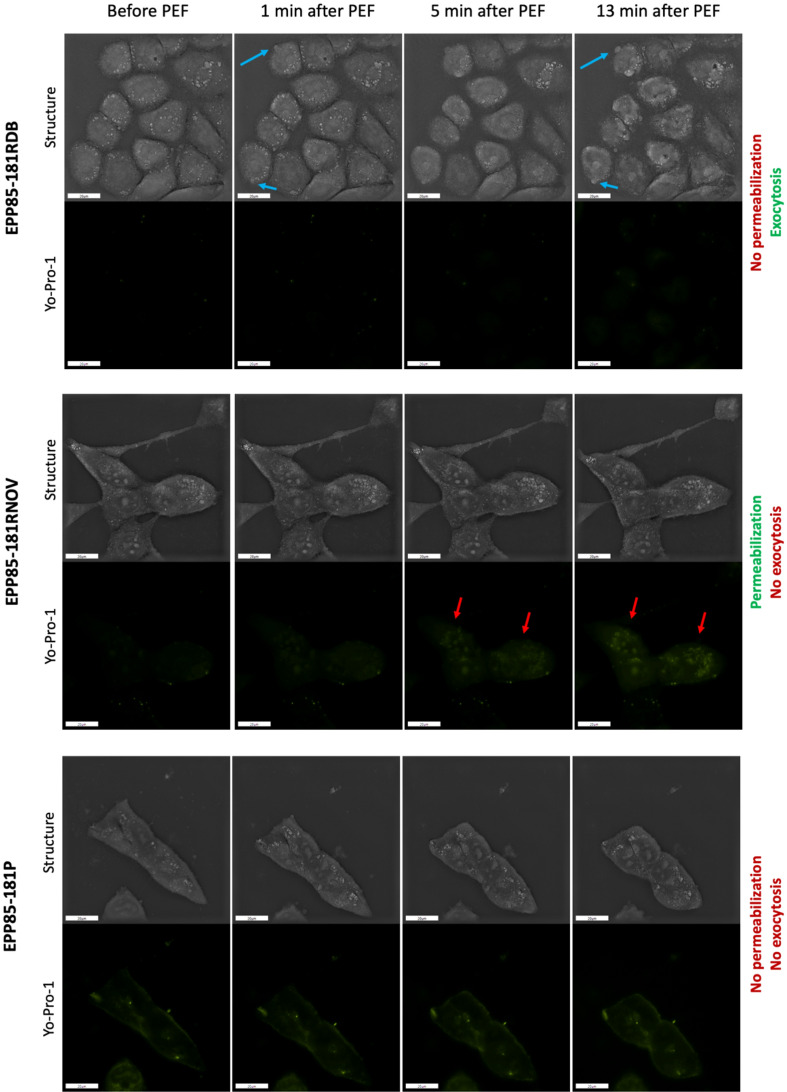
The response of EPP85-181RDB, EPP85-181RNOV and EPP85-181P cell lines to nsPEF (8 kV/cm, 200 ns, 100 pulses, 10 kHz). The photographs were captured before, 1 min, 5 min and 13 min after pulse application. The top panel shows the ultrastructure of the cells obtained by the holotomography reconstruction and the bottom panel shows the fluorescence of Yo-Pro-1 after the administration of nsPEF, blue arrows—MVs release from the cell membrane, red arrows—YoPro-1 stain signal in the cells.

To analyze the effects of nsPEF on the morphology of pancreatic cancer cells we implemented the holotomographic microscopy. Figure 1 presents the response of cells towards nsPEF. Depending on the cell line, we observed MVs release, membrane permeabilization and cell shrinkage following nsPEF treatment. EPP85-181RDB—the smallest cells underwent extensive MVs release, which was detectable 5 min after a stimulation with the electric field. Small, vesicular structures could be observed nearby the cell membrane minutes after nsPEF treatment. The most extensive MVs release occurred 13 min after the PEF treatment. 24 h post experiment we continued to observe the detached vesicles surrounding the cells. No Yo-Pro-1 inflow was observed during the experiment. In contrast, in the significantly larger, EPP85-181RNOV cells, permeability to Yo-Pro-1 dye was greatly improved by nsPEF application. Aside from the high level of fluorescent dye uptake, no morphological changes were observed in this cell line. EPP85-181P cells did not experience detached membrane vesicles nor underwent permeabilization. In response to PEF treatment, the EPP85-181RNOV cells size was reduced and they may presumably became resistant to damage.

Molecular dynamics simulations were conducted to investigate different membrane effects of nsPEF (Table 1). Simulations of the electric field action on cell membranes were performed on models with various surface tension and cholesterol content. By reducing the surface tension from 0 to −30 mN/m we obtained the invagination of the membrane model. These data suggest that the surface tension plays an important role in cellular response to PEFs. When the surface tension remains low, low voltage nsPEF does not evoke electroporation. Instead, invagination may occur in the membrane regions low in cholesterol. Consequently, changes may be observable such as the release of MV’s detected in EPP85-181RDB cell line. In contrast, when the same voltage is applied on a membrane when the surface tension remains high triggers membrane electroporation, which seems to be the case for EPP85-181RNOV cells line.

**Table 1:**
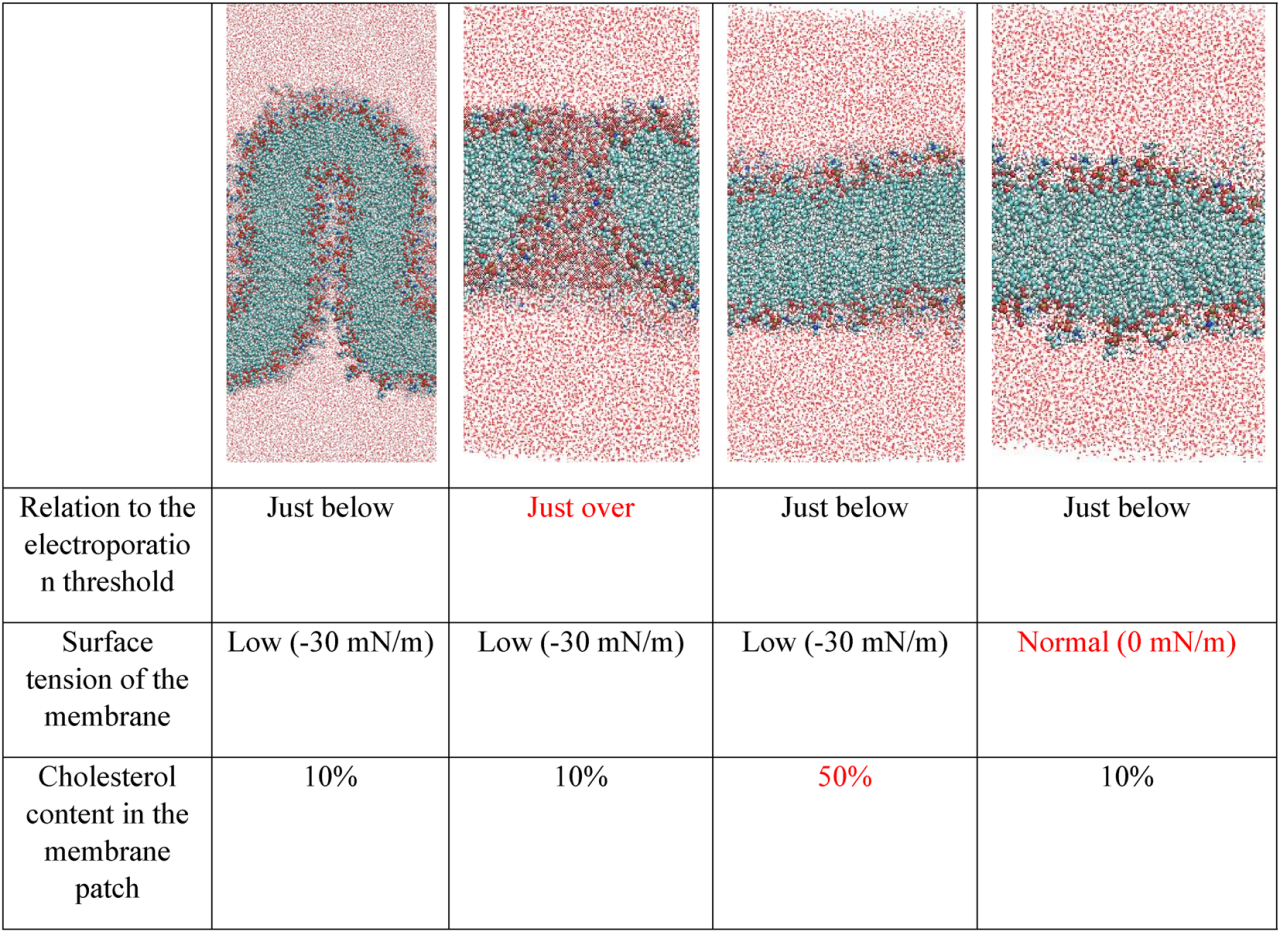
Molecular dynamics simulations concerning the effects of electric field on the morphology of the membrane with respect to the electroporation threshold, surface tension of the membrane and cholesterol content in the membrane patch.

**Figure 2:**
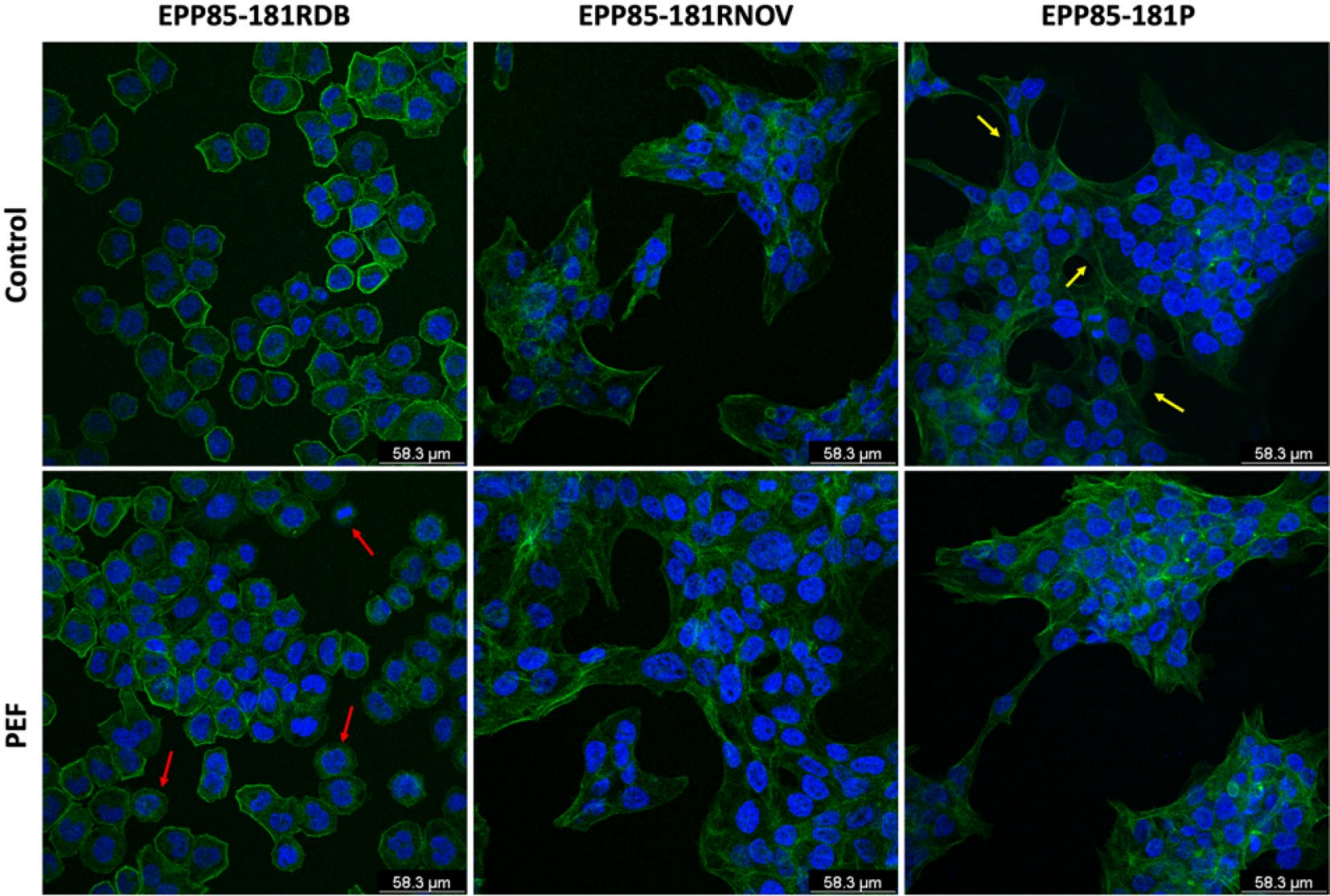
Fluorescent staining of F-actin (green) and nuclei (blue), red arrows show the loss of peripheral cytoskeleton in EPP85-181RDB cells after PEF stimulation, yellow arrows point the presence of protruding extremities before stimulation of EPP85-181P cells with PEF; nuclei (blue). Confocal microscope. Scale bar: 58.3 µm.

Figure 2 presents the organization of F-actin 24 h after PEF treatment. Confocal laser microscopy studies of actin remodeling in EPP85-181RDB cells, show high vulnerability of their cytoskeleton to PEFs. After PEF treatment, the number of cells lacking a peripherally aligned cytoskeleton had increased (Fig. 2). The reduction in peripheral actin organization may be correlated with the release of microvesicles from the membrane of the cells. Conversely, cells which underwent membrane permeabilization (EPP85-181RNOV) present almost no changes in actin organization following the nsPEF treatment (Fig. 2). The subtle changes in F-actin cytoskeleton following 24 h incubation after nsPEF treatment indicate reversibility of the electroporation process. It was also observed that nsPEF-treated EPP85-181P cells have significantly less protrusion structures in comparison to control cells (Fig. 2, yellow arrows). Interestingly, these changes concerned only protrusions involved in cellular adhesion, but not those involved in cell communication.

### Effects of nsPEF on multidrug resistance

**Figure 3:**
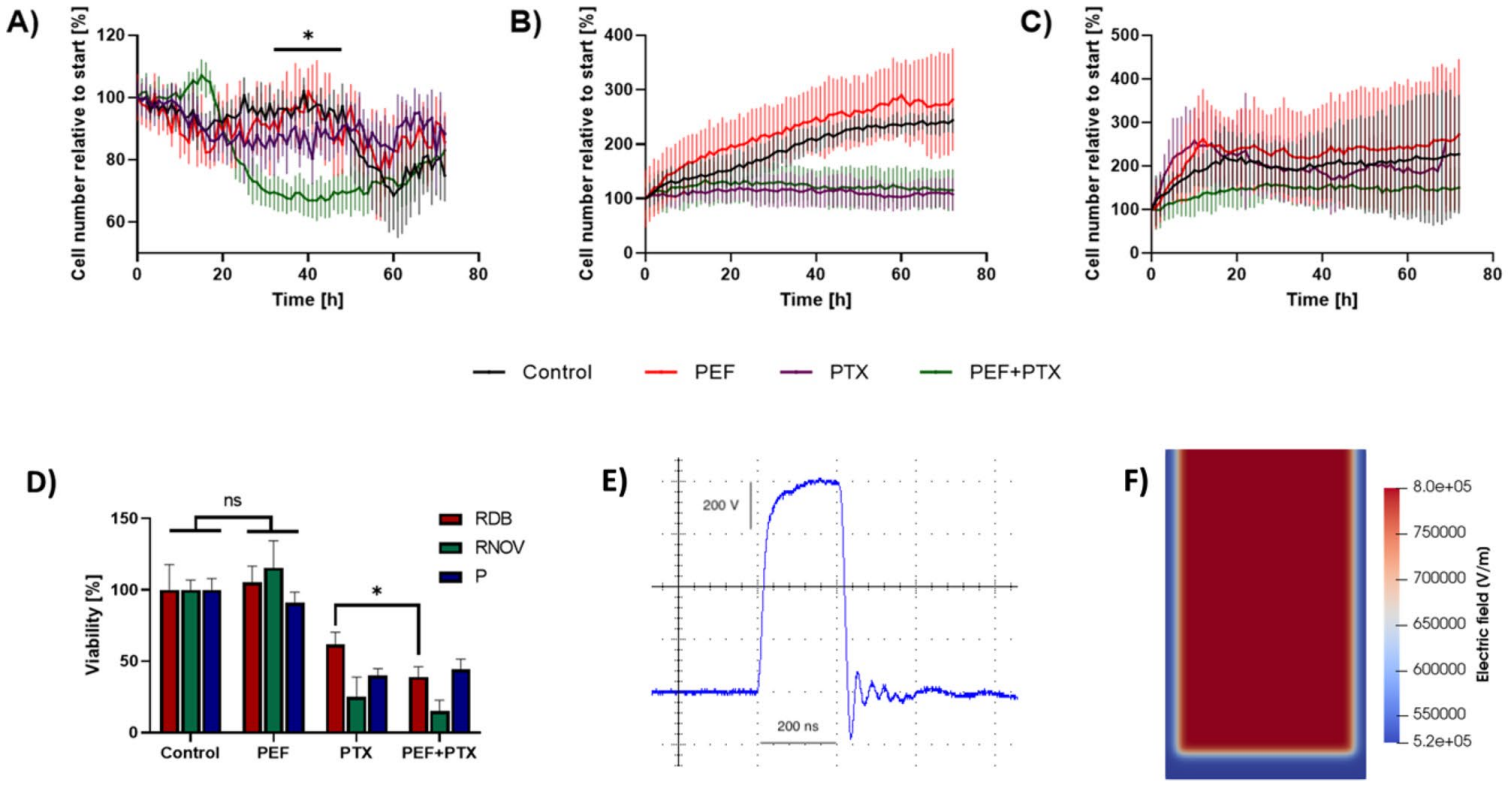
(**A–C**) The relative number of EPP85-181RDB (**A**), EPP85-181RNOV (**B**), EPP85-181P (**C**) cells as the function of time measured after the administration of 50 nM paclitaxel alone and in combination with nsPEF. The study was conducted using CellcyteX automated cell counter and analyzer, ANOVA * p < 0.05, Black—Control, Red—PEF, Purple—Paclitaxel, Green—Paclitaxel added 24 h after PEF treatment; (**D**) Presto Blue assay after the exposure of the EPP85-181RDB, EPP85-181RNOV and EPP85-181P cells to 50 nM paclitaxel combined with nsPEF. The drug was added to the cells 24 h after nsPEF treatment and the cells were left for 48 h in the drug 50 nM solution, ANOVA *p < 0.05; (**E**) oscilloscope caption of nsPEF of 200 ns, 100 pulses, 10 kHz and 8 kV/cm; (**F**) computer modelling of the electrotherapy cuvette (1 mm gap), which shows an even distribution of the field inside the cuvette. Slight differences are observed in the periphery of the chamber.

After analyzing the structural alternations in pancreatic cancer cells subjected to nsPEF (see the pulses on Fig. 3E), we examined the modulatory effects of PEF on the sensitivity of cancer cells to chemotherapy. For the study we did not consider the electro-transport of the drug through the cell membrane, but rather we wanted to focus on the long-term changes in cells MDR proteins and its consequences on the sensitivity of cancer cells to chemotherapy. The expression of MDR proteins and susceptibility of the cells to paclitaxel was examined 24 h after nsPEF, which is much longer than the time required for membrane resealing after electropermeabilization^14,28,29^. Figures 3A–C report the changes in the number of EPP85-181RDB, EPP85-181RNOV and EPP85-181P cells following (1) the application of PEFs only, (2) incubation with 50 nM paclitaxel and (3) combination of PEFs with 50 nM paclitaxel. The drug was added to the cell culture 24 h after PEF treatment and the cells were monitored in real time. For EPP85-181RNOV and EPP85-181P cell lines, Presto Blue viability assay revealed no differences in cells’ viability after 48 h standalone incubation with paclitaxel and the combination of PEF with paclitaxel (Fig. 3D). The only cell line, which became more vulnerable to paclitaxel after treatment with nsPEF was EPP85-181RDB (Fig. 3A,D). It is worth noting that the effects were transient (visible only up to ~ 48 h after incubation with paclitaxel) and gradually became negligible in 60 h time. EPP85-181RNOV cells were highly susceptible towards paclitaxel and indeed the drug inhibited their proliferation (Fig. 3B). The effect of paclitaxel observed on EPP85-181P cell line was negligible (Fig. 3C). As for the monitoring of the external factors, which may affect the cell during nsPEF treatment, we performed the computer modelling simulations of electric field distribution inside the electroporation cuvette. We found that there is not an extensive heat nor other type of radiation inside the chamber. Besides, the electric field inside the simulation box is very stable and even inside the cuvette (Fig. 3F).

**Figure 4:**
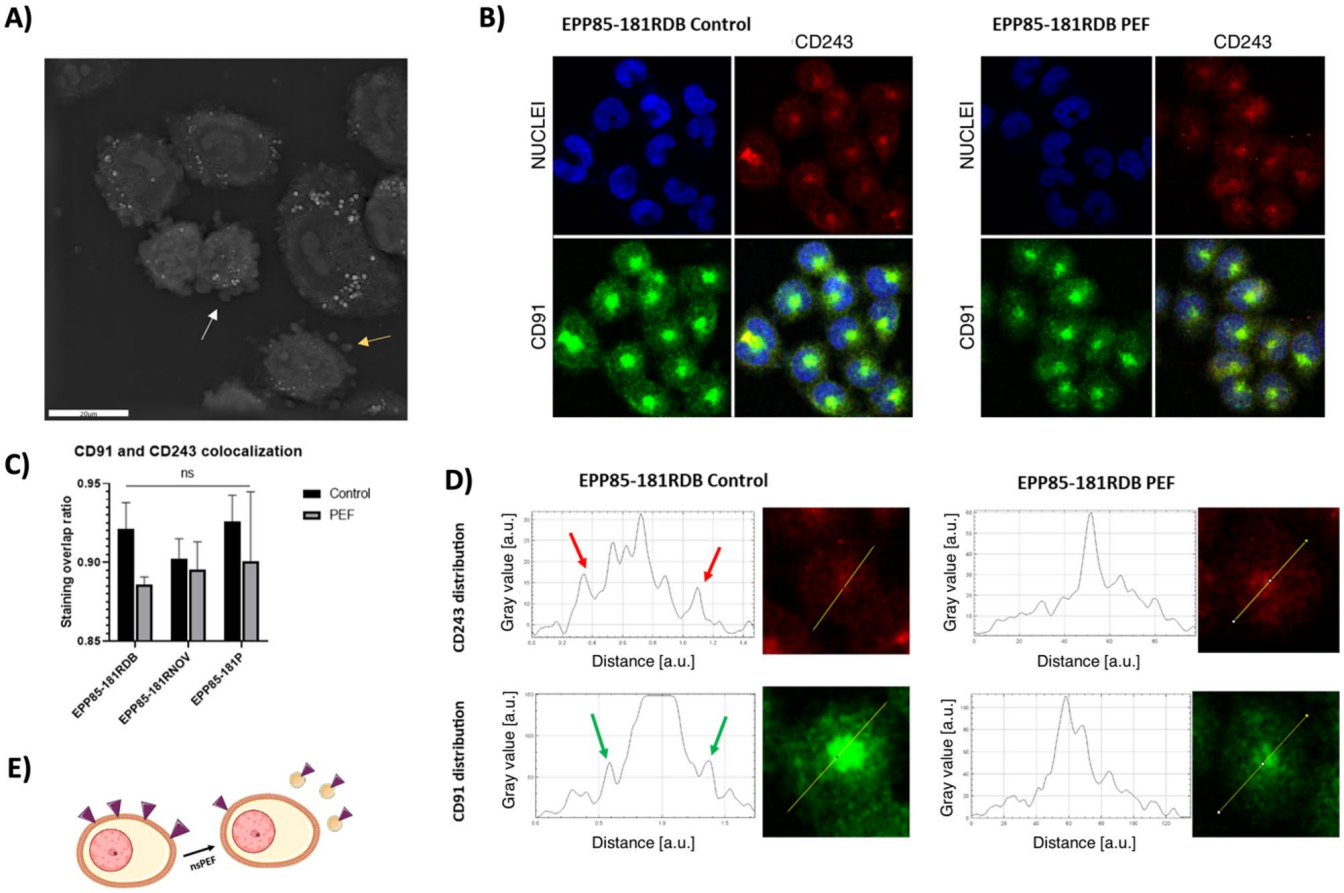
(**A**) Holotomographic microscopy photograph showing EPP85-181RDB cells 13 min after nsPEF treatment. White arrow indicates micro vesicles (MV’s) being detached from the cell membrane and yellow arrow shows already freely detached MV’s from the membrane; (**B**) the immunofluorescence staining of CD91 (green) and CD243 (red) 24 h following the exposure to nsPEF treatment of EPP85-181RDB cells; (**C**) colocalization studies between CD91 and CD243 signal shift 24 h after the cells were exposed to nsPEF, analyzed with LasX software; (**D**) signal distribution plots in the EPP85-181RDB cells exposed to nsPEF, the photographs were analyzed in ImageJ software via the distribution plot module; (**E**) proposed mechanism in which, the cells reduce the membrane content of MDR proteins via the release of MDR proteins containing vesicles.

Our holotomographic microscopy studies showed the release of microvesicles from EPP85-181RDB cells subjected to nsPEF. Figure 4A presents the mechanism in which cell detaches small portion of its membrane (depicts as the white arrow) and releases micro vesicles outside the cells (yellow arrow). Chemotherapy-sensitizing effect of PEF in EPP85-181RDB cells was further examined with the confocal microscopy (Fig. 4B). The fluorescence studies of P-gp (CD243) and LRP (CD91) on EPP85-181RBD showed high abundance of the proteins present in a single spot of the cell 24 h after the nsPEF treatment. By analyzing the distribution of the signal in the cells, we may conclude that the membrane content of both MDR proteins highly decreases after treatment with nsPEF (Fig. 4D). The release of microvesicles from the cell membrane of EPP85-181RDB cells alters the expression of MDR proteins localized on the plasmalemma. The process results in the dimmed signal of MDR proteins in confocal microscopy (Fig. 4D). Colocalization studies of both CD91 and CD243 showed no signal overlap and confirmed that the highest difference in the immunoreactivity may be detected in EPP85-181RDB cells (Fig. 4C). The mechanism connecting the release of microvesicles and the loss of membrane proteins is depicted on the Fig. 4E.

### Effects of nsPEF on spheroids’ growth and cell-to-cell interactions

**Figure 5:**
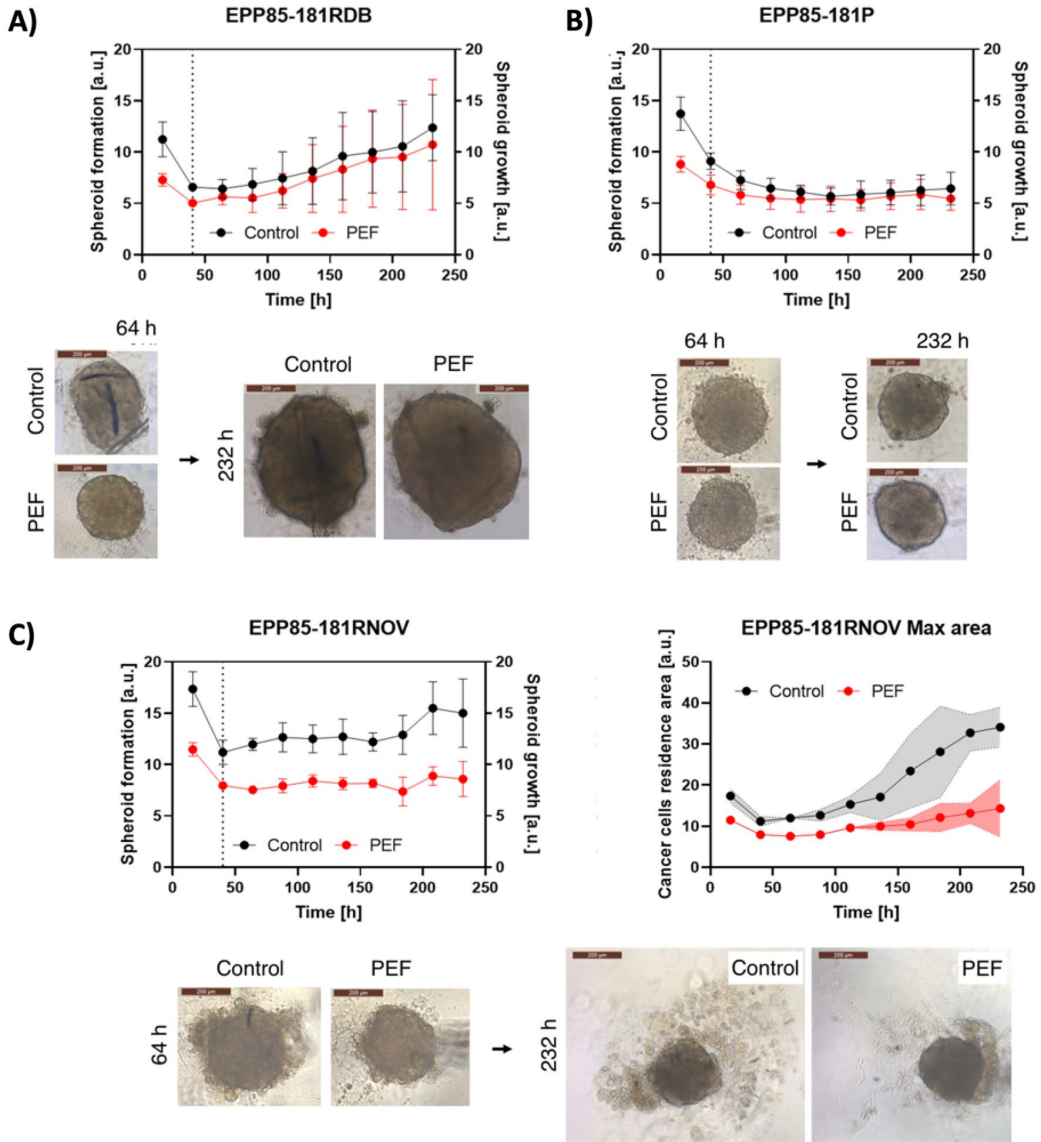
Kinetic studies concerning the growth of 3D cell culture formed by EPP85-181RDB (**A**), EPP85-181P (**B**) and EPP85-181RNOV (**C**) cell lines supported by the photographs of the formed spheroids. The plot is divided into the spheroid formation phase and spheroid growth phase, separated with a vertical line; right side of (**C**) shows the maximum area occupied by the cancer cells.

**Figure 6:**
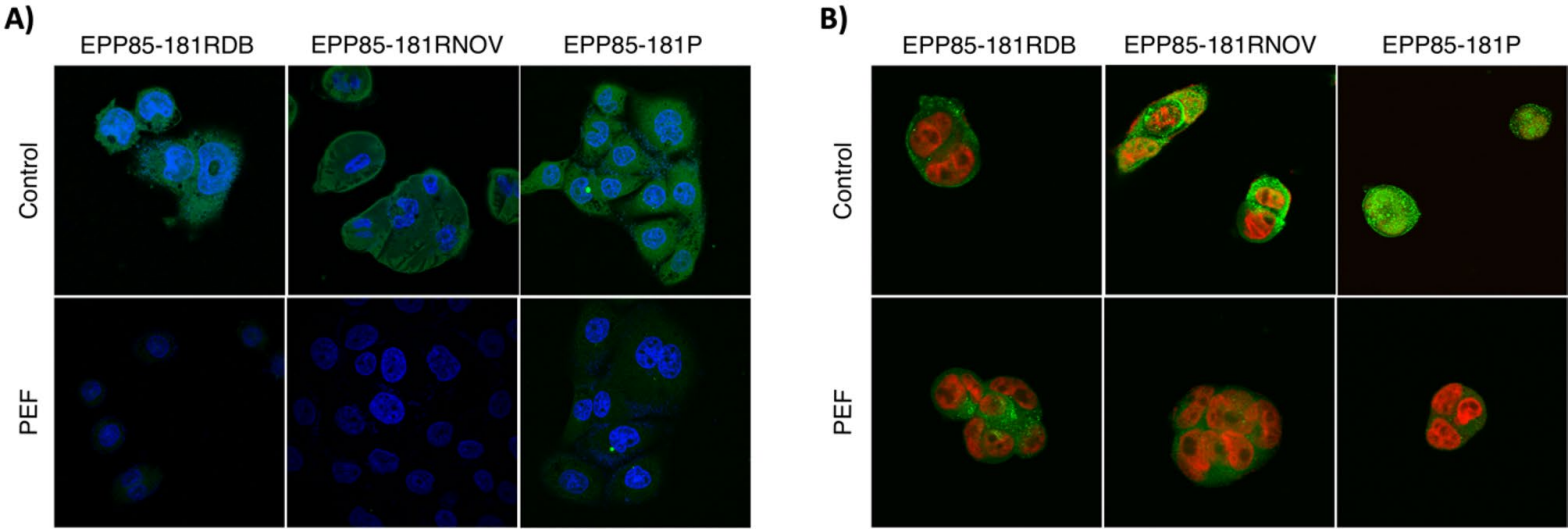
(**A**) Immunodetection of N-cadherin (green) expression pattern 24 h after nsPEF treatment in pancreatic cancer cells (blue—nuclei); confocal microscope; (**B**) immunodetection of pan-cadherin expression pattern (green) 24 h after nsPEF treatment in pancreatic cancer cells (red—nuclei).

The last aspect of the study focused on the effects of nsPEF on the adhesive properties of the pancreatic cancer cells as well as the formation and growth of 3D cell culture spheroid. With EPP85-181RDB cells, we observed changes in the formation of the spheroid solely within the first two days after treatment with electric field (Fig. 5A). However, it should be noted that this cell line’s spheroids are less stable than other cell lines, which led to higher standard deviation of the spheroid’s size compared to other cell lines (Fig. 5B,C). After the spheroid formation was complete, we started the observation of its growth. There was no significant size difference between PEF-treated and control EPP85-181RDB spheroids. Initially, EPP85-181RDB derived spheroids were about 300 μm in diameter, EPP85-181RNOV were about 500 μm and EPP85-181P were the smallest with about 250 μm in diameter (Fig. 5A–C). Interestingly, the size of EPP85-181RDB cell-mass after PEF was not significantly different from control. Moreover, as visualized with microscope, PEF-treated cells were more adherent to each other and formed irregularly aligned cell groups. The confocal microscopy studies revealed a partial reduction of the fluorescent signal from pan-cadherin and shift of the signal from the cells’ periphery to the center (Fig. 6A) Unlike pan-cadherin, the signal of N-cadherin the fluorescence was almost undetectable (Fig. 6B). Noteworthy, membrane signal after PEF treatment was highly reduced in comparison to the control sample.

EPP85-181RNOV (second analyzed pancreatic cancer cell line) cells responded to treatment with nsPEF by forming significantly reduced spheroids throughout the observation period (Fig. 5C). Moreover, the 3D culture composed from cells treated with nsPEF were more densely attached to each other after 100 h following the experiment. The pancreatic cancer cells did not escape from the cell-mass to the environment in comparison to the control cells and the spread of the cells was observed at a much lower extent in the samples treated with the nsPEF. Interestingly, the EPP85-181RNOV cells formed similar cells’ conglomerates to EPP85-181RDB cells, but the expression of the pan-cadherin was much lower than in the control samples (Fig. 5C). Similarly to EPP85-181RDB, the cells also lacked the membrane signal of the protein.

The third analyzed pancreatic cancer cell line—EPP85-181P reduced the size of 3D cell culture in the first hours following PEF treatment. However, after longer incubation times, the cancer spheroid was comparable to control spheroids (Fig. 5B). In addition, the cells reduced the fluorescence signal of pan-cadherin and aggregated 24 h following the nsPEF treatment (Fig. 6B). Even after treatment, the N-cadherin signal was still detected but highly reduced both in the cytoplasm and on the cell membrane (Fig. 6A).

## Discussion

The anticancer potential of nsPEF was evaluated within the scope of two main applications—electrochemotherapy and irreversible electroporation^30,31^. Electrochemotherapy utilizes electric pulses which do not permanently disrupt the cell membrane, but rather pulses which reseal after the introduction of the drug to the cytoplasm of the targeted cell. In contrast, irreversible electroporation permanently disrupts the cell integrity, which is the most prominent cell death factor in this therapy approach. The effectiveness of cell membrane permeabilization and cell death induction as a function of the frequency of the delivered pulses, their length, number and the applied voltage has been addressed^21,31^. Cited studies showed that not all pulse types induce cell permeabilization and may accordingly be applied to transport the drugs throughout cell membranes. For instance, Kiełbik et al. proved that nsPEF induces electropermeabilization under high applied voltage and MHz frequency of pulses delivery. Other study by Szlasa et al. proved that irreversible electroporation occurs when using extremely long 10 ms pulses in microsecond electroporation protocols^32^. The other application of nsPEF in the anticancer therapy is irreversible electroporation, which is triggered by extremely high electric fields and high frequencies of pulse delivered^30,32^. In contrast to microsecond pulses, nsPEFs need to be delivered at much higher (10 times standard) electric field intensities to observe similar permeabilization effects^21,32^. When electroporation is applied to treat cancer tissues, most of the affected cells are those which lay between the electrodes. However, when the electric field is high, cancer cells outside the electrodes’ range are also subjected to low electric field intensities. Interestingly, cells which are not in between electrodes can also be affected by PEFs application. This phenomenon is called a “Bystander effect”^33^. To our knowledge, no thorough studies were carried out which characterized molecular changes in cells in close proximity to sub-electroporation threshold electric fields range.

In our study three analyzed pancreatic cancer cell lines responded differently to the same parameters of the electric field. These differences may arise from the variation of surface tension of the cell membranes. Interestingly, based on Schwan’s equation, cell size does not solely correlate with the electroporation threshold for the nanosecond pulses^34^. Therefore, the differences in the organization of actin fibers and cells’ protrusions may be responsible for the differences in the surface tension of all pancreatic cancer cells. EPP85-181P cells were not permeable to Yo-Pro-1 following the nsPEF treatment; however, the actin-containing filopodia were no longer visible actin fibers were remodeled. The shrinkage and fibers reorganization may be involved in the higher resistance of the cells towards PEF. Reparation mechanisms following PEF treatment are surely involved in EPP85-181P response to PEF due to the retained viability of the cells following PEF treatment. In contrast, EPP85-181RNOV cells did not see an alternation of the organization of F-actin fibers which resulted in permeabilization of the membrane. EPP85-181RDB cells responded differently—by the release of microvesicles (MV) and the reorganization of actin fibers, the cells may avoid the disruption of the cell membrane during nsPEF treatment. MVs release was not similar to previously described blebbing, which leads to cell apoptosis^32,35,36^. The size of vesicular structures released from the cells is much smaller in contrast to the apoptotic release of blebs^35,37^. Interestingly, the formation of MVs was not instantaneous but occurred 5 to 13 min after the exposure. This time required for the cells’ response suggests that the cell membrane is not the only cell element involved in the process, but complex reorganization of the cytoskeleton occurs as well. Indeed, in EPP85-181RDB cells, peripherally aligned F-actin fibers were disrupted following the nsPEF treatment, which reduced the surface tension of the membrane. The surface tension is also affected by the lipid composition of the cancer cell membrane^38^. Our molecular dynamics simulations showed that the reduced surface tension of the membrane leads to its invagination of the membrane and further the MVs release. Therefore, membrane permeabilization in EPP85-181RNOV cells rises from the lack of molecular response to sheer stress induced by the electric field.

Given that the electric field affects the release of MVs from EPP85-181RDB cells, we investigated the effects of the nsPEF treatment on the expression of CD91 and CD243 membrane proteins from the MDR protein family. Our study revealed that the treatment reduces membrane content of both proteins in EPP85-181RDB cells. Co-localization of CD91 and CD243 proteins is pronounced for the EPP85-181RDB cell line, whereas in the other cell lines, the difference was not visible. Interestingly, in none of the cell lines was there a statistically significant difference. The alternations in membrane content of the protein may arise from the release of MVs from the membrane. CD91/LRP is both a multidrug resistance protein and a common component of endocytosis vesicles^39^, as it is involved in clathrin-dependent endocytosis^40^. On the other hand, CD243 is a membrane MDR protein^41^ responsible for the regulation of protein trafficking to the membrane during P-gp biosynthesis^42^. No study proving the presence of CD243 could be found, therefore, the higher content of CD91 in membrane vesicles may give rise to a slightly altered colocalization pattern of both proteins. Our data suggests that the release of MDR proteins from the cell membrane is related to the release of MVs and may solely reduce the cell resistance to paclitaxel chemotherapy. This effect was observed only for EPP85-181RDB cell line, 48 h after exposure to the nsPEF and was supported by the Presto Blue viability assay. Within three days of the study, the cells regained their resistance to chemotherapy and thus the positive effects of PEFs diminished.

The last part of the study concerned the effects of nsPEF on the 3D cell culture models. This study analyzed the kinetics of formation of a 3D cell culture model and next its growth dynamics and adhesive properties of the cancer cells. In general PEFs induced dimming and shifting of the distribution of the cadherins from the cell membrane and the cytoplasm, toward sole localization in the cytoplasm of the cell. Pan-cadherin targeting antibody is specific towards E-, N-, R- and P-cadherins. The antibody targets the junction proteins, which connects the cells to each other^43,44^. On the other hand, the N-cadherin fluorescence studies indicated a loss of proteins from the membrane, which according to Yan et al. might be responsible for the reduced growth of the cells in spheroid models^45^. Other papers have shown that targeting N-cadherin with monoclonal antibodies or silencing of the N-cadherin gene leads to a reduced proliferation of cancer cells and a lower cell migration^46^. Interestingly, the content of N-cadherin in pancreatic cancer cells was almost completely reduced, while other cadherins belonging to pan-cadherin group were present to some extent after PEF treatment. Gathered data suggest that the cells retain their adhesive properties, simultaneously reducing proliferation and migration due to the lack of N-cadherin. Xiao He Li et al. study shows that cadherins interact with cytoplasmic F-actin, preventing cells from tearing apart^47^. This stays in agreement with our results which prove the involvement of the interactions between cadherin and F-actin in the response of the cells to the stress factor. Interestingly, Plestant et al. presented the evidence that adhesive interactions of N-cadherin limit the recruitment of microtubules to cell–cell contacts through the organization of actomyosin^48^. Han et al. contributed to the topic by presenting vinculin as the key regulator of the cadherin-actin cellular mechanoreceptor system^49^. Cadherin-actin complex has to be resistant to external tension to retain proper function. F-actin disruption should dysregulate the cell adhesive properties and junction interactions between the cells^50,51^. Peripheral polymerization of actin results in the formation of the protrusions of the cells. Protrusions are responsible for the lateral movement of cell membranes to the neighboring cells to keep cadherins in contact. Therefore the disruption of F-actin cytoskeleton after nsPEF treatment may result in the impaired adhesive function of cells^52^. Taking all of this into consideration, we suggest that nsPEF reduce the cadherin signal from the cell surface and simultaneously impair the functioning of the junctions between cells. Impaired cadherins cannot be involved in the movement of the nearby cells, which reduces cancer growth and mobility. It is worth investigating why the response of EPP85-181RNOV cells is stable and the effect in two other cell lines diminish within the first days after the experiment. It may be due to the fact that only EPP85-181RNOV cells did not reorganize the actin fibers in response to nsPEF, but rather suffered from membrane electroporation and the reorganization of cadherins with little effect on actin fibers. It is likely that in the other two cell lines, the cadherin-actin complexes are retained while in EPP85-181RNOV cells, the complexes are disrupted and unable to reconstitute.

The effects of nsPEF on pancreatic cancer cells depend on the surface tension of the membrane. The effects vary, but in all the cell lines the effects are always positive. Overall, nsPEF delivery led to (i) the reduction in cancer formation and growth (ii) cell sensitization to the following chemotherapy. Due to the inequity of the effects, nsPEF treatment in our proposed protocol should not be considered as the standalone anticancer treatment, but rather as a positive effect observed in the margin of the tumor accompanying the irreversible electroporation of the cancer tissue. All the data from the project was summarized in Table 2.

**Table 2:** Summary of the molecular effects of nsPEF on pancreatic cancer cells (+ slight effect, ++ moderate effect, +++ high effect).

	EPP85-181RDB	EPP85-181RNOV	EPP85-181P
Permeabilization	No	Yes	No
Membrane response	Release of microvesicles	No morphological change	Cell shrinkage
Actin response	Lack in the periphery	No visible effect	Reorganization
CD91 and CD243 colocalization	Most variable	Colocalized	Colocalized
Effect of nsPEF on chemotherapy resistance	Yes	No	No
Long lasting attenuation of cancer growth	No	Yes	No
Induction of cells adherence	+	++	+
Loss of cadherin	+++	+++	++

## Conclusions

Effects of nsPEF on various pancreatic cancer cells are divergent and vary with respect to the surface tension of the cell membrane as well as the applied voltage. The alignment of the F-actin in the cell regulates the response of the membrane to nsPEF and three options remain possible—electropermeabilization, cell shrinkage or the release of microvesicles. The latter reduces the membrane content of the MDR proteins and may induce the transient increase in the susceptibility to chemotherapy. In case of the adhesive properties of the cells, the positive effect of nsPEF may be observed in all pancreatic cancer cell lines. Namely, the disruption of cadherin-actin interaction restricts the growth of 3D cell culture model of the cancer and prevents the detachment of the cancer cells from the cell mass. Low voltage nsPEF exerts positive therapeutic effect on cancer cells, but the extension of the process relies on the surface tension of the membrane.

## Materials and methods

### Cell culture

The study was conducted on three human-derived cell lines from patients with pancreatic ductal adenocarcinoma: resistant to daunorubicin (EPP85-181RDB), resistant to mitoxantrone (EPP85-181RNOV) and parental (EPP85-181P). For the study, we chose the chemotherapy resistant cell lines with high expression of MDR proteins. The primary culture cell lines were kindly shared by Dr. H. Lage (Charité University Hospital, Institute of Pathology, Berlin, Germany). The cultures were maintained at 37 °C under high humidity in the automated CO_2_ incubator (Binder). Cells were cultivated in the modified Leibovit’z (L-15) medium (Gibco, Life Technologies, Carlsbad, CA) supplemented with 10% fetal bovine serum, 1% antibiotics (100 IU/ml penicillin and 0.1 mg/ml streptomycin), 1.5% sodium bicarbonate (7.5%, Gibco), 0.1% glucose (45%, Sigma), 2.5 mM ultraglutamine (Lonza, Basel, Switzerland), 0.2% insulin (10 mg/mL, Sigma) and 30 TIU/L aprotinin (BioShop, Canada). Cells were collected from flasks using Trypsin (ThermoFisher Scientific).

### Spheroid 3D cell culture

The cells were detached from flasks and resuspended in Phosphate EP buffer (pH  7.4, 1 mM MgCl2 and 250 mM sucrose). The experiments were performed on 2 × 10^6^ cells/ml concentration. Next, the cells were resuspended in L-15 medium. Afterwards, the cells were carefully counted in Kova’s chambers and further, 5000 cells were seeded in each well of Grainer Facilitate 96-well 3D cell culture plates. The cells were captured under the standard cell culture conditions. After 40 h of incubation, we examined the formation of the spheroids. Each day from then, we captured photos of the spheroids on light microscope. Further analysis included the calculation of the microtumor size and area.

### PEF treatment

50 μl of cells in the concentration of 2 × 10^6^/ml, suspended in 10 mM phosphate buffer (pH  7.4, 1 mM MgCl_2_ and 250 mM sucrose, were placed in 1 mm cuvettes with aluminum electrodes (BTX, Syngen Biotech, Poland). The square wave electroporator (100 ns to 1 ms) developed in the Institute of High Magnetic Fields (VGTU, Vilnius, Lithuania) was used to deliver electric pulses. The cells were exposed to 8 kV/cm × 200 ns × 100 pulses delivered at 10 kHz frequency (0.085 J, 1.6 kJ/L). Pulse shapes and amplitudes were controlled with an MDO3052 oscilloscope (Tektronix, Beaverton, OR). Subsequently, the cells were suspended in a culture medium and placed in 96 or 6 well plates on cover glasses.

### Drug preparation

Paclitaxel (Abcam, 10 mg, ab120143) solutions were prepared from the DMSO stock solutions of 5 µM. The required concentrations were achieved with a dilution of stock in EP buffer to 50 nM concentration. Drug was added to the cells 24 h after nsPEF treatment to avoid transporting the drug through the cell membrane. This attempt was made to analyze the vulnerability of the cells to paclitaxel and not its transport through the membrane. 24 h was considered sufficient time for the closure of reversible pores and fixation of nsPEF-induced changes in the cell membrane.

### Holotomographic microscopy

Live holotomography was performed using a 3D Cell Explorer microscope (Nanolive SA, Ecublens, Switzerland). Pancreatic cancer cells were imaged using 35 mm Ibidi glass-bottom μ-Dish dishes (Ibidi GmbH, Germany). During imaging, temperature was set to 37 °C and controlled using an Ibidi Heating System (Ibidi GmbH, Germany), while the sufficient amount of CO_2_ was maintained by using a CO_2_-independent culture medium (Sigma-Aldrich). Initially, we placed the electroporation needles surrounding the visualized cell groups. Afterwards, we started to recorded a video and after a short while treated cells with PEFs. The snapshots were taken each 1 min for at least 13 min. Further, the data from the microscope was gathered and analyzed using STEVE Software.

Concerning the Yo-Pro-1 dye uptake studies, the cells were permeabilized according to the description of permeabilization of cell suspension. However, before PEFs delivery, the green-fluorescent YO-PRO-1 stain (Y3603, Thermo Fisher Scientific, Waltham, MA) in the concentration of 1 μl/ml was added to EP buffer. YO-PRO-1 cellular uptake reflects the degree of plasma membrane’s permeabilization. The fluorescence of YO-PRO-1 was excited with 488 nm wavelength and measured on Nanolive 3D Cell Explorer microscope.

### Molecular dynamics simulation of membrane response

The molecular dynamics simulations were performed with GROMACS 2018.3 software^53^ on the calculational cluster in the Department of Theoretical Chemistry and Physics at the Lorraine University. The models for simulations were built with CHARMM-GUI web software and visually inspected with VMD software^54,55^. The simulated systems were composed of a membrane in the ionic water solution. The membrane was composed of ~ 256 lipids per membrane layer. All the systems were built considering mixture of lipids-cholesterol and 1-palmitoyl-2-oleoyl-sn-glycero-3-phosphocholine—POPC. Two systems representing 10% and 30% cholesterol were modeled, each with POPC. Before the simulation, the system was solvated in physiological conditions of NaCl water (TIP3) solution. Six (5 mM) calcium ions were present in each system. Both water compartments were separated by the introduction of the vacuum above and below the system. The whole simulation was performed in the periodic boundary conditions. The simulation proceeded with the CHARMM36 force field^54^. The systems were minimized, equilibrated (100 ns, NPT conditions: Nose–Hoover thermostat and Berendsen barostat) and simulated for 10 ns in the NVT ensemble to obtain the value of the surface tension in the air–water interphase. Afterwards, the system was simulated for 100 ns under various ionic gradient conditions. The gradient was introduced by moving the ions throughout the membrane. The electroporation simulation was carried under NPγT (constant surface tension, number of molecules, pressure and temperature) conditions. Moreover, we reduced the surface tension of the membrane in one of the studied systems. After the simulation, the system was evaluated if the pore was formed during the time of the simulation and the visual representations of the membrane were presented on the layouts.

### Confocal microscopy

To visualize the actin filaments, pan-cadherin, N-cadherin and nuclei of the cells, an Olympus Fluoview FV1000 confocal laser scanning microscope (Olympus) was used. confocal microscope was used. Control and nsPEF-treated cells were seeded on microscopic glass plates. Following a 24 h of incubation, the cells were fixed with a 4% formaline for 10 min and washed three times with PBS. Afterwards, the cells were incubated with 5% FBS in 4% Triton X-100 diluted in PBS to block the unspecific interactions in further steps. Actin filaments were stained with Invitrogen™ Alexa Fluor™ 488 Phalloidin (2 μg/ml, Life Sciences—Thermo Fisher Scientific) according to the manufacturer protocol. Fluoroshield™ with DAPI (4,6-diamidino-2-phenylindole, Sigma-Aldrich) was applied to visualize the nuclei and to mount the cells. Primary antibodies were used: mouse monoclonal anti-CD91, PE, eBioscience™ diluted (1:200) in 4% Triton X-100 in PBS, mouse monoclonal anti-CD243, PerCP-eFluor™, eBioscience™ (ThermoFisher Scientific) diluted in 4% Triton X-100 in PBS (1:200), rabbit polyclonal anti-pan-Cadherin (Invitrogen) diluted (1:300) in 4% Triton X-100 in PBS, and rabbit monoclonal anti-N-cadherin (Invitrogen) diluted (1:300). All antibodies were diluted with a 4% Triton X-100 in PBS. As a secondary antibody, anti-rabbit antibody was used conjugated with AlexaFluor488™ diluted (1:250) in 4% Triton X-100 in PBS was used. For the imaging, an Olympus Fluoview FV1000 confocal laser scanning microscope (Olympus) was used. The images were recorded by employing the Plan-Apochromat 60× objective. Any contrast and brightness adjustments were performed in FV10-ASW_Viewer or in ImageJ. The images were analyzed with the use of ImageJ to evaluate the expression pattern of the proteins in the cross-section of the cells.

### Electric field distribution

The spatial electric field distribution has been simulated in OpenEP software. A two-dimensional model of the cuvette electrodes (aluminum, 1 mm gap) a has been designed and the conductivity of the surrounding medium (phosphate buffered saline (PBS)) of 1.5 S/m has been selected. The 8 kV/cm electric field distribution was simulated and presented in the visual way.

### Viability assays

For Presto Blue viability studies we have added the reagent in 1:10 (reagent to culture medium) ratio to each well and fluorescence was measured, after 30 min of incubation at 37 °C, using a GloMax Discover microplate reader (Promega; Exc. 520 nm/Em. 580–640 nm). The results were expressed as the percentage of viable cells relative to untreated control cells.

### CellcyteX studies

CellcyteX live-cell imaging system acquires phase contrast images, which are analyzed with detection software. Detection software was used to determine cell viability by measuring cell confluency and comparing the results with cell count and nuclei studies. The system was used to assess the kinetics of cells’ growth after treatment with nsPEF and Paclitaxel. In the study we aimed to establish the optimal time for the viability tests and examine if the antiproliferative effect of nsPEF is permanent. Control and PEF-treated cells were seeded on 96-well plates. After 24 h at 37 °C paclitaxel was added to wells and cells were further incubated at 37 °C in the incubator set to work with CellcyteX operating Cellcyte Studio software. The experiment was performed in replicates and each well consisted of four, square zones, in which the cells were counted. Further, the images were analyzed with Aivia Software. The results were expressed as the percentage of viable cells relative to untreated control cells.

### Statistical analysis

The experiments were performed in at least 3 replicates. Data were expressed as mean ± SD and analyzed by one-way ANOVA (in GraphPad Prism 8), with p < 0.05 being considered statistically significant.

## Data Availability

The datasets generated during and analyzed during the current study are available on request to the authors.
